# Plasma metabolites associated with arterial stiffness in patients with type 2 diabetes

**DOI:** 10.1186/s12933-020-01057-w

**Published:** 2020-06-11

**Authors:** Naoto Katakami, Kazuo Omori, Naohiro Taya, Shoya Arakawa, Mitsuyoshi Takahara, Taka-aki Matsuoka, Hiroshi Tsugawa, Masahiro Furuno, Takeshi Bamba, Eiichiro Fukusaki, Iichiro Shimomura

**Affiliations:** 1 Department of Metabolic Medicine, Osaka University Graduate School of Medicine, 2-2, Yamadaoka, Suita, Osaka 565-0871 Japan; 2 Department of Metabolism and Atherosclerosis, Osaka University Graduate School of Medicine, Osaka, Japan; 3 Laboratory of Bioresource Engineering, Department of Biotechnology, Graduate School of Engineering, Osaka University, Osaka, Japan; 4 Department of Diabetes Care Medicine, Graduate School of Medicine, Osaka University, Osaka, Japan; 5 RIKEN Center for Sustainable Resource Science, Yokohama, Kanagawa Japan; 6 Division of Metabolomics, Medical Institute of Bioregulation, Kyushu University, Fukuoka, Japan

**Keywords:** Arterial stiffness, Atherosclerosis, Brachial ankle pulse wave velocity (PWV), Diabetes, Metabolomics

## Abstract

**Background:**

Although an increased arterial stiffness has been associated with traditional coronary risk factors, the risk factors and pathology of arterial stiffness remain unclear. In this study, we aimed to identify the plasma metabolites associated with arterial stiffness in patients with type 2 diabetes mellitus.

**Methods:**

We used the metabolomic data of 209 patients with type 2 diabetes as the first dataset for screening. To form the second dataset for validation, we enlisted an additional 31 individuals with type 2 diabetes. The non-targeted metabolome analysis of fasting plasma samples using gas chromatography coupled with mass spectrometry and the measurement of brachial-ankle pulse wave velocity (baPWV) were performed.

**Results:**

A total of 65 annotated metabolites were detected. In the screening dataset, there were statistically significant associations between the baPWV and plasma levels of indoxyl sulfate (r = 0.226, p = 0.001), mannitol (r = 0.178, p = 0.010), mesoerythritol (r = 0.234, p = 0.001), and pyroglutamic acid (r = 0.182, p = 0.008). Multivariate regression analyses revealed that the plasma levels of mesoerythritol were significantly (β = 0.163, p = 0.025) and that of indoxyl sulfate were marginally (β = 0.124, p = 0.076) associated with baPWV, even after adjusting for traditional coronary risk factors. In the independent validation dataset, there was a statistically significant association between the baPWV and plasma levels of indoxyl sulfate (r = 0.430, p = 0.016). However, significant associations between the baPWV and plasma levels of the other three metabolites were not confirmed.

**Conclusions/interpretation:**

The plasma levels of indoxyl sulfate were associated with arterial stiffness in Japanese patients with type 2 diabetes. Although the plasma levels of mannitol, mesoerythritol, and pyroglutamic acid were also associated with arterial stiffness, further investigation is needed to verify the results.

## Background

Cardiovascular disease (CVD) is one of the major causes of death and impairment of quality of life in patients with diabetes and its risk in these patients is severalfold higher compared to the general population.

Recently, several studies have indicated that arterial stiffness, which depends on the structural and geometric properties of the arterial wall, plays a critical role in the pathogenesis of atherosclerosis and cardiovascular diseases (CVD) [1–3]. Therefore, the prevention of arterial stiffness via the clarification of its risk factors and pathophysiology would contribute to reducing the morbidity and mortality of CVD.

It has been revealed that various metabolic abnormalities are involved in the development of arterial degeneration and cardiac dysfunction. Notably, the alterations of glucose homeostasis, not only in diabetes but also prediabetes, play crucial roles [4, 5]. The alterations in the vascular homeostasis due to endothelial and vascular smooth muscle cell dysfunction, one of the main features of atherosclerosis, are induced by a prolonged exposure to hyperglycemia and insulin resistance clustering with other risk factors such as obesity, arterial hypertension, and dyslipidemia. The major biochemical pathways involved in this process are presumed to be the overproduction of reactive oxygen species, the increased formation of advanced glycation end-products (AGEs) and activation of the AGEs-RAGE axis, polyol and hexosamine flux, protein kinase C activation, and chronic vascular inflammation [6, 7]. However, the details of metabolic abnormalities related to the development of atherosclerosis, especially arterial stiffness, in patients with diabetes remain unclear.

In this study, we aimed to identify the plasma metabolites associated with arterial stiffness in patients with type 2 diabetes, using gas chromatography coupled with mass spectrometry (GC/MS)-based non-targeted metabolomics.

## Methods

### Subjects

This study employed two independent datasets. As the first dataset for screening, we used the metabolomic data of 209 individuals with type 2 diabetes, which were obtained from the previous study that aimed to evaluate the association between the metabolites and the presence of coronary artery disease [8] (Additional file 1: Figure S1). In brief, the subjects with type 2 diabetes based on the criteria of the Japan Diabetes Society [9] and age 20–74 years were consecutively recruited at the Osaka University Medical Hospital. Those individuals who were (1) in the perioperative period or with a serious infection or injury, or (2) experiencing severe renal dysfunction or end-stage renal failure (serum creatinine > 2.0 mg/dL) were excluded. Taken together, a total of 209 patients who had been measured for brachial-ankle pulse wave velocity (baPWV) were included into the present study, among 240 patients who were enrolled into the original study between June 2014 and October 2016. Subsequently, as the second dataset for validation, we recruited an additional 31 patients with type 2 diabetes who met the same eligibility criteria at Osaka University Hospital between November 2017 and February 2019.

The study protocol was approved by the Research Ethics Committee of Osaka University Hospital (approval number 13454 and 16374) and the study was conducted in accordance with the principles of the Declaration of Helsinki. Written informed consent was obtained from all the subjects after they received a complete explanation of the study.

### Clinical and biochemical analysis

The following clinical data such as age, sex, body mass index, cardiac risk factors, prior cardiac disease, and medication were obtained from each patient at entry. Fasting blood samples were collected and HbA1c, serum lipids, and creatinine levels were measured using standard laboratory protocols [10]. At the same time, the fasting plasma used for metabolomics analysis was collected, cooled immediately in a freezer at 4 °C, and subsequently, stored at − 80 °C.

The determination of hypertension (defined as systolic blood pressure (SBP) ≥ 130 mmHg; diastolic blood pressure (DBP) ≥ 80 mmHg; or the use of anti-hypertensive medication), dyslipidemia (defined as serum LDL cholesterol ≥ 3.1 mmol/L (120 mg/dL); serum TG ≥ 1.7 mmol/L (150 mg/dL); HDL-cholesterol ≺ 1.0 mmol/L (40 mg/dL); or lipid-lowering medication use), and obesity (body mass index ≥ 25 kg/m^2^) were based on the criteria of the Japan Diabetes Society [11]. The estimated-glomerular-filtration-rate (eGFR mL/min/1.73 m^2^) was calculated using an equation proposed by the Japanese Society of Nephrology [12].

### Assessment of arterial stiffness

In the present study, the level of arterial stiffness was assessed using baPWV, which reflects the stiffness of both the aorta and peripheral arteries in an arm and a leg. The measurement of baPWV was performed using the same volume-plethysmographic apparatus as for ABI (BP-203RPE II form PWV/ABI), with subjects in the supine position after at least 5 min of rest, as previously reported [13–17]. Precisely, four oscillometric cuffs, each connected to a plethysmographic sensor that determined volume pulse from and to an oscillometric pressure sensor that measured blood pressure, were wrapped on both the brachia and ankle; two electrocardiogram electrodes were placed on each wrist. The cuffs were simultaneously pressurized to the approximate value of the patient’s diastolic pressure such that the pulse volume waveforms could be recorded using semiconductor pressure sensors. The distance between the sampling points of baPWV was calculated automatically according to the height of the subject. The path length from the suprasternal notch to the ankle (La) was calculated as: La = 0.8129*height (in cm) + 12.328. The path length from the suprasternal notch to the brachium (Lb) was calculated as: Lb = 0.2195*height − 2.0734. The baPWV was calculated according to the following formula: baPWV = (La − Lb)/Tba, where Tba refers to the time interval between the wave front of the brachial waveform and that of the ankle waveform [14]. Two simultaneous measurements of baPWV were recorded on the right side and left side, respectively, and the higher value of these readings was used as the representative value for each individual.

### Metabolic profiling analysis of plasma samples

The measurement of samples in the first and the second cohorts were performed independently. The details are described in the Additional file 1. In brief, 50 µL of plasma was mixed with 150 µL of deaerated H_2_O containing 0.2 mg/mL of ribitol, which was used as an internal standard, and subsequently was preprocessed. After randomizing the sample sequence, the metabolic profiling analysis of the preprocessed sample was conducted on a Shimadzu TQ8040 GC system (Shimadzu Corporation, Kyoto, Japan) that was connected to a mass spectrometer. The obtained GC/MS data were converted to the Analysis Base File (ABF) format using the ABF converter (https://www.reifycs.com/AbfConverter/index.html). The feature detection, spectra deconvolution, metabolite identification, and peak alignments were performed using the MS-DIAL software ver. 2.72 (http://prime.psc.riken.jp/Metabolomics_Software/MS-DIAL/index.html) [18]. Each metabolite was calibrated using the LOWESS/Spline correction curve based on the quality control (QC) values [19], after the substances for which the residual standard deviation (RSD) of QC samples was above 40% were excluded from further analysis. As a result of these processes, the intra- and inter-day precision of the quantification of each metabolite was secured within a certain range. In addition, the variability of quantification of each metabolite was corrected.

### Statistical analysis

The clinical data are reported as the mean ± standard deviation (SD) for continuous variables and percentages for dichotomous variables.

An association of two variables was analyzed using the Pearson’s correlation. Furthermore, the association between detected metabolites and baPWV after adjusting for clinical risk factors (age, sex, HbA1c, smoking history, hypertension, hyperlipidemia, and obesity) was evaluated by multivariate regression analyses. The log-transformed value of each metabolite was used for association analysis.

The comparisons between two groups were assessed with the Mann–Whitney’s U-test for continuous variables.

The prediction ability of the metabolites to identify arterial stiffness (baPWV ≥ 1800 cm/s) was examined by the receiver-operating-characteristic (ROC) curve analyses.

For all tests, a p-value < 0.05 was considered as statistically significant. These statistical analyses were performed using SPSS version 26 (SPSS Inc., Chicago, IL, USA).

## Results

The clinical characteristics of the study subjects that composed the screening dataset and those of validation dataset are shown in Table 1.

**Table 1:** Clinical characteristics of study subjects

	The first dataset	The second dataset
Number of subjects	209	31
Age (years)	60.5 ± 11.5	63.9 ± 10.5
Male (sex) (n, %)	121 (57.9)	13 (41.9)
Diabetes duration (years)	13.9 ± 9.9, n = 206	16.8 ± 11.1
HbA1c (%)	9.0 ± 1.9	9.1 ± 2.1
FPG (mg/dL)	154 ± 53, n = 205	145 ± 46
Obesity (n, %)	134 (64.1)	21 (67.7)
BMI (kg/m^2^)	27.2 ± 5.4	26.7 ± 5.1
Hypertension (n, %)	138 (66.0)	22 (71.0)
Systolic BP (mmHg)	126 ± 18	129 ± 17
Dyslipidemia (n, %)	137 (65.6)	26 (83.9)
Total cholesterol (mg/dL)	198 ± 50	201 ± 61
HDL cholesterol (mg/dL)	49 ± 15	53 ± 12
LDL cholesterol (mg/dL)	114 ± 39	120 ± 49
Triglyceride (mg/dL)	182 ± 143	157 ± 97
eGFR (mL/min/1.73 m^2^)	73 ± 24	71 ± 21
Smoking history (n, %)	109 (52.2), n = 208	15 (48.4)
Medication use		
Diabetes (n, %)	178 (85.2)	26 (83.9)
Hypertension (n, %)	127 (60.8)	19 (61.3)
Dyslipidemia (n, %)	99 (47.4)	22 (71.0)
baPWV (cm/s)	1728 ± 384	1813 ± 275

Data are presented as mean ± standard deviation (SD) for continuous variables and percentage for dichotomous variables

*BMI* body mass index, *FPG* fasting plasma glucose, *BP* blood pressure, *GFR* glomerular filtration rate

It is notable that a total of 65 annotated metabolites were detected from plasma samples after excluding the metabolites for which RSD of QC samples was more than 40% from the screening dataset consisting of a total of 209 patients with type 2 diabetes. Firstly, the candidate biomarkers for arterial stiffness were screened by analyzing the association of these 65 metabolites with baPWV. There were statistically significant associations between the baPWV and the plasma levels of indoxyl sulfate (r = 0.226, p = 0.001), mannitol (r = 0.178, p = 0.010), mesoerythritol (r = 0.234, p = 0.001), and pyroglutamic acid (r = 0.182, p = 0.008) (Table 2, Additional file 1: Figure S2). A stepwise multivariate regression analysis including only the metabolites with a significant p value (< 0.05) after univariate analysis revealed that the plasma levels of mesoerythritol (β = 0.198, p = 0.005) and indoxyl sulfate (β = 0.150, p = 0.034) were significantly associated with baPWV.

**Table 2:** Univariate association of plasma levels of metabolites and baPWV in dataset 1

Metabolites	r	p value
1,5-Anhydro glucitol	0.111	0.108
1-Hexadecanol	0.080	0.251
2-Aminobutyric acid	0.004	0.953
2-Aminoethanol	0.053	0.447
2-Hydroxybutyrate	− 0.061	0.380
2-Hydroxypyridine	0.005	0.939
3-Amino isobutyric acid	0.072	0.302
Alanine 2TMS	0.052	0.459
Alanine 3TMS	0.063	0.367
Allose + Mannose	− 0.024	0.735
Asparagine	0.018	0.798
Cholesterol	0.117	0.091
Creatinine	0.024	0.726
Fructose	0.089	0.200
Galactose + Glucose	0.001	0.991
Gluconic acid	− 0.039	0.577
Glucose	− 0.003	0.961
Glucuronate	0.062	0.375
Glutamic acid	− 0.007	0.921
Glutamine	0.129	0.063
Glyceric acid	0.001	0.987
Glycine	0.097	0.164
Glycolic acid	0.006	0.931
Histidine	0.023	0.744
Hydroxyproline	− 0.084	0.229
Hypoxanthine	− 0.033	0.638
Indoxyl sulfate	*0.226*	*0.001*
Inositol	0.100	0.151
Isocitric acid + Citric acid	0.090	0.197
Isoleucine 1TMS	0.052	0.452
Isoleucine 2TMS	− 0.006	0.928
Lactic acid	0.027	0.695
Lauric acid	0.112	0.107
Leucine 1TMS	0.029	0.678
Leucine 2TMS	− 0.050	0.475
Lysine	− 0.026	0.708
Mannitol	*0.178*	*0.010*
Mannose	− 0.031	0.653
Mesoerythritol	*0.234*	*0.001*
Methionine	0.096	0.165
Myristic acid	0.116	0.094
Nonanoric acid (9:0)	0.042	0.543
Oleic acid (18:1n-9)	0.032	0.649
O-Phosphoethanolamine	0.43	0.532
Oxalacetic acid + Pyruvate	0.084	0.225
Palmitic acid (16:0)	0.095	0.172
Palmitoleic acid (C16:1n7)	0.062	0.371
Phenylalanine	0.067	0.333
Phosphate	− 0.117	0.091
Proline	− 0.007	0.917
Psicose + Tagatose	0.031	0.657
Pyroglutamic acid	*0.182*	*0.008*
Quinic acid	0.097	0.164
Serine 2TMS	0.072	0.302
Serine 3TMS	0.048	0.486
Stearic acid (18:0)	0.080	0.250
Sucrose	− 0.063	0.368
Threonic acid	− 0.061	0.384
Threonine 2TMS	0.045	0.517
Threonine 3TMS	0.034	0.624
Tryptophan	− 0.029	0.675
Tyrosine	0.039	0.578
Urea	0.132	0.056
Uric acid	− 0.054	0.439
Valine	− 0.038	0.581

The Pearson’s correlation coefficient was evaluated to detect the metabolites associated with the baPWV

Italic font indicates statistically significant (p < 0.05) differences

Multivariate regression analyses revealed that the plasma levels of mesoerythritol were significantly associated with baPWV (β = 0.163, p = 0.025) and that of indoxyl sulfate were marginally associated with baPWV (β = 0.124, p = 0.076), even after adjusting for the conventional clinical risk factors such as age, sex, HbA1c, smoking history, hypertension, hyperlipidemia, obesity, and eGFR. However, the plasma levels of mannitol and pyroglutamic acid were not associated with baPWV, after adjusting for clinical risk factors (β = 0.067, p = 0.400 and β = 0.067, p = 0.325, respectively).

The prediction abilities of indoxyl sulfate, mannitol, mesoerythritol, and pyroglutamic acid estimated by the area under the ROC curve were 0.637 (95% CI 0.558–0.715, p = 0.001), 0.644 (95% CI 0.564–0.724, p < 0.001), 0.659 (95% CI 0.583–0.734, p < 0.001), and 0.595 (95% CI 0.515–0.675, p = 0.020), respectively. The highest sensitivity and specificity were obtained when the cut-off values of log transformed indoxyl sulfate, mannitol, mesoerythritol, and pyroglutamic acid were set at 7.052 (sensitivity 0.64, specificity 0.63), 7.303 (sensitivity 0.58, specificity 0.70), 8.320 (sensitivity 0.53, specificity 0.73), and 10.451 (sensitivity 0.41, specificity 0.76), respectively.

**Fig. 1:**
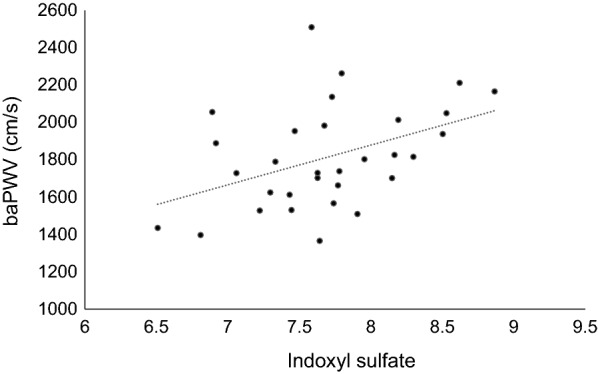
Association between the plasma levels of indoxyl sulfate and baPWV. The plasma levels of indoxyl sulfate were correlated with baPWV in the independent validation dataset consisting of a total of 31 patients with type 2 diabetes (r = 0.430, p = 0.016)

In the independent validation dataset consisting of a total of 31 patients with type 2 diabetes, there was a statistically significant association between the baPWV and the plasma levels of indoxyl sulfate (r = 0.430, p = 0.016) (Fig. 1). However, there was no statistically significant association between the baPWV and the plasma levels of mannitol (r = 0.043, p = 0.817), mesoerythritol (r = 0.250, p = 0.175), and pyroglutamic acid (r = − 0.243, p = 0.187) (Additional file 1: Figure S2).

The direct comparison using the Mann–Whitney’s U-test revealed that the plasma levels of indoxyl sulfate (p = 0.037), mannitol (p < 0.001), and mesoerythritol (p = 0.007) were also significantly higher in subjects with CVD (n = 50) as compared to those without CVD (n = 159). The multivariate logistic regression analyses showed that the plasma level of mannitol was significantly associated with CVD even after adjusting for the clinical risk factors such as age, sex, HbA1c, smoking history, hypertension, hyperlipidemia, and obesity (Odds ratio per 1U increment [95% CI] 1.84 [1.25–2.73], p = 0.002); however, the plasma levels of indoxyl sulfate and mesoerythritol were not associated with CVD.

## Discussion

In this study, we evaluated the association between the plasma metabolites assessed by GC/MS and arterial stiffness measured with baPWV in patients with type 2 diabetes and revealed that the plasma levels of indoxyl sulfate, mannitol, mesoerythritol, and pyroglutamic acid were associated with baPWV. Furthermore, among them, the plasma levels of mesoerythritol were significantly associated and that of indoxyl sulfate were marginally associated with baPWV, even after adjusting for traditional cardiovascular risk factors. In addition, the substantial association between indoxyl sulfate and baPWV was confirmed in the independent validation dataset. However, the prediction abilities of these metabolites estimated by the area under the ROC curve were not high, suggesting that there remain large hurdies before they can be used as a biomarker for diabetic macroangiopathy in the clinical setting.

The baPWV, a quantitative indicator for arterial stiffness, is linked to the traditional CV risk factors including diabetes and has been confirmed as a promising predictor of coronary artery disease [20], myocardial injury [21], and future cardiovascular events [22]. It has been reported that the indices of chronic hyperglycemia, insulin resistance [23], postprandial hyperglycemia [24], and daily glucose fluctuations were associated with arterial stiffness [25]. Oxidative stress and inflammation are presumed to play important roles in the progression of arterial stiffness in the subjects with alterations of glucose homeostasis. Circulating lipid-related metabolites such as lysophosphatidylcholines and lysophosphatidylethanolamines were also reported to be associated with oxidative stress, inflammation, arterial stiffness in Chinese men with newly diagnosed type 2 diabetes [26]. Recently, Jung et al. reported that an age-related increase in the plasma lactosylceramide is an independent predictor of an increased arterial stiffness in middle-aged individuals and subjects progressing to impaired fasting glucose [27, 28]. It is still cumbersome to draw a conclusion as to whether the plasma levels of lysophospholipids and/or lactosylceramide are also associated with baPWV in patients with prolonged type 2 diabetes, as we did not measure these metabolites.

There have been several reports on the study of non-targeted metabolomics to identify the novel metabolites associated with arterial stiffness in the general population. Li et al. performed a non-targeted metabolomics profiling among 1239 participants of the Bogalusa Heart Study and identified 16 metabolites associated with arterial stiffness (4 for augmentation index and 12 for PWV) independent of traditional risk factors. Among them, the metabolites selected in our study namely indoxyl sulfate, mannitol, mesoerythritol, and pyroglutamic acid were not included [29]. However, it is possible that the diabetic status may behave as an effect modifier of the association between the metabolites and arterial stiffness; thus, significant association may only be apparent among subjects with type 2 diabetes mellitus. Indeed, in the PREDIMED trial, a cardiovascular prevention trial testing the mediterranean diet, positive association between the lysine pathway metabolites and the incidence of CVD was observed in subjects with type 2 diabetes not in non-diabetic subjects [30].

It is notable that several previous studies would be useful for the discussion of the mechanism of how the selected metabolites could be associated with the progression of atherosclerosis. Indoxyl sulfate, a metabolite of dietary protein or tryptophan, is a circulating uremic toxin. Since this was the sole metabolite that was associated with baPWV in both the original and the validation dataset, it would be the most promising biomarker for arterial stiffness among the metabolites we evaluated in this study. Notably, experimental studies demonstrated that indoxyl sulfate directly accelerates the progression of atherosclerosis by several mechanisms, such as the inhibition of endothelial function and increase of smooth muscle cell proliferation [31, 32]. Furthermore, several clinical studies indicated the associations between the circulating levels of indoxyl sulfate and the atherosclerosis-related phenotypes. It has been reported that this molecule may contribute to the development of CVD in subjects with chronic kidney disease [33]. Additionally, higher concentrations of plasma indoxyl sulfate were associated with an increase in the carotid IMT in chronic CAD patients with preserved renal function [34]. We have also reported that higher concentrations of plasma indoxyl sulfate were associated with increased carotid IMT and presence of CAD in patients with T2DM [8]. Thus, the result of the current study is consistent with previous reports, suggesting that the elevation of plasma indoxyl sulfate levels could accelerate atherosclerosis. Notably, renal dysfunction may not be the sole reason for the high plasma concentrations of uremic toxins. Recently, the gut microbiota has been increasingly considered as a relevant source of uremic toxins [35–37]. Indole is produced by intestinal bacteria as a degradation product of tryptophan and is subsequently absorbed and metabolized in the liver to indoxyl sulfate. As diabetes affects the gut microenvironment and is associated with a distinct gut microbial composition and metabolism [37, 38], it could also be related to the elevation of plasma indoxyl sulfate levels.

Mannitol and mesoerythritol, also known as sugar alcohols, occur widely in nature and have a similar chemical structure. Although there have been several reports on the effect of these metabolites on atherosclerosis, they showed controversial results [39–42]. A few in vivo or in vitro studies reported that both mannitol and mesoerythritol acted as antioxidants, suggesting their anti-atherosclerotic function [39, 40]. On the other hand, another study showed that mannitol induces endothelial cell apoptosis [41]. Similarly, it was reported that the exposure to mesoerythritol aggravated cerebral ischemic injury in mice and impaired the function of the endothelial progenitor cells [42]. However, since the plasma mannitol level was no longer associated with baPWV after adjustment for traditional risk factors for atherosclerosis, its association with arterial stiffness could be a reflection of confounding relationships with the factor correlation.

Pyroglutamic acid is a cyclic derivative of glutamine. In vitro studies demonstrated that pyroglutamate is associated with monocyte chemoattractant protein-1 activity and the expression of HUVEC adhesion factor [43]. Furthermore, Shiomi et al. comprehensively analyzed the serum of WHHLMI rabbits that spontaneously developed coronary atherosclerosis and reported that the serum levels of pyroglutamic acid were significantly associated with the development of coronary atherosclerosis [44]. These findings suggest that pyroglutamic aci could be a novel biomarker of atherosclerotic disease.

The limitations of our study should be discussed. First, the sample size was insufficient to draw conclusions. In the first step, we performed multiple statistical analyses, which may have generated false-positive results derived from multiple testing. Therefore, in the second step, we confirmed the associations between the metabolites and arterial stiffness using an independent population. However, the sample size of the second step (n = 31) was too small to demonstrate weaker associations between two variables where the Pearson’s correlation coefficient was under 0.36. As a result, although a statistically significant association between baPWV and the plasma levels of indoxyl sulfate (r = 0.430, p = 0.016) was confirmed, significant associations between baPWV and the plasma levels of the other three metabolites (e.g. mannitol, mesoerythritol, and pyroglutamic acid) were not confirmed. Thus, the statistical power was not sufficient for the detection of weak associations as well as drawing conclusions. Therefore, the results should be taken as preliminary data. Further investigation with a large study population is required to identify the metabolites that might have been overlooked due to poor statistical power and confirm the possible associations that were observed in the present study. Second, no conclusion as to whether there are causal relations between the detected metabolites and arterial stiffness could be drawn from this cross-sectional study. Future prospective cohort studies and experimental studies using in vivo or in vitro models will be necessary to elucidate this point. Third, the association between baPWV and the plasma levels of indoxyl sulfate, mannitol, or pyroglutamic acid did not reach statistical significance after adjusting for conventional clinical risk factors, suggesting that the direct impact of these metabolites on arterial stiffness is limited, if any. It is also noted that all the participants of this study were Japanese. The Japanese population has historically shown low levels of LDL-C, while a combination of economic recovery and a shift toward more a westernized lifestyle and habits have been accompanied by a gradual increase in serum cholesterol in the recent years [45]. As a result, the Japanese population shows a consistent trend toward relatively low incidence of CVD, which is closely related to LDL-C, as compared to people in the United States and European countries. Therefore, it would be not be possible to generalize our findings to non-Japanese populations. Finally, although the GC/MS analysis is able to measure diverse classes of compounds in a sensitive manner, this approach cannot fully cover the entire metabolome of a biological sample. A combination with other MS analyses would enable the investigation of metabolome profiles with a broader coverage. Notwithstanding these limitations, our study indicates that the detected metabolites could be associated with arterial stiffness in patients with type 2 diabetes.

## Conclusions

The plasma level of indoxyl sulfate was associated with arterial stiffness in Japanese patients with type 2 diabetes. Although the plasma levels of mannitol, mesoerythritol, and pyroglutamic were likely to be associated with arterial stiffness, further investigation with a large study cohort is needed to verify the results.

## Supplementary information

**Additional file 1. Figure S1.** Disposition of study subjects. **Figure S2.** Association between the plasma levels of indoxyl sulfate, mannitol, mesoerythritol, and pyroglutamic acid, and baPWV in the first and the second datasets.

## Data Availability

The datasets generated and/or analyzed during our study can be obtained from the corresponding author on reasonable request.

## Abbreviations

baPWV: Brachial-ankle pulse wave velocity
CVD: Cardiovascular disease
DBP: Diastolic blood pressure
FPG: Fasting plasma glucose
GC/MS: Gas chromatography coupled with mass spectrometry
GFR: Glomerular filtration rate
eGFR: Estimated glomerular filtration rate
QC: Quality control
RSD: Residual standard deviation
SBP: Systolic blood pressure
UAE: Urinary albumin excretion
