# Supplementary material for: Plasma metabolites associated with arterial stiffness in patients with type 2 diabetes
Source: Cardiovasc Diabetol. 2020 Jun 11;19:75. doi: 10.1186/s12933-020-01057-w (PMC7291560; doi:10.1186/s12933-020-01057-w)

**Supplementary Materials**

**Detailed method of metabolic profiling analysis of plasma samples**

The measurement of samples of the first and the second cohort were performed totally independently. At first, 50 µL of plasma was mixed with 150 µL of deaerated H_2_O containing 0.2 mg/mL of ribitol, which was used as an internal standard, (1200 rpm, 10 min, 4°C). Then, 800 µL of deaerated MeCN was added (1200 rpm, 10 min, 4°C) and centrifuged (16000×*g*, 3 min, 4°C). Next, 300 µL of supernatant were transferred to an Eppendorf tube. All the steps up to this point were performed in a cold room at 4°C. The samples were then dried in a vacuum centrifuge dryer for 1 hour and lyophilized overnight. For derivatization, first, 100 µL of methoxyamine hydrochloride in pyridine (20 mg/mL) was added to the samples, and the mixture was incubated (1200 rpm, 90 min, 30°C). A second derivatizing agent, *N*-methyl-*N*-trimethysilyl-trifuroacetamide (MSTFA), was then added and the mixture was incubated (1200 rpm, 30 min, 37°C). After centrifucation (16000×*g*, 3 min), 100 µL of derivatized samples were transferred to glass vials.

The metabolic profiling analysis of the preprocessed sample was conducted on a Shimadzu TQ8040 GC system (Shimadzu Corporation, Kyoto, Japan) that was connected to a mass spectrometer. The samples (1 µL each) were injected into the GC/MS system in split mode (split ratio 1:25). InertCap 5MS/NP capillary column (GL sciences INC, Tokyo, Japan) was used and helium gas flow rate through the column was set at 1.12 ml/min. The column temperature was set to 80°C for 2 min and then raised to 330°C for 12 min. The temperature of the transfer interface and ion source was set to 310°C and 280°C, respectively. The selected mass range was set to 85-500 m/z with electron impact ionization (70 eV).

All plasma samples were analyzed after randomizing sample sequence over multiple batches. A quality control (QC) sample generated by mixing the same volume of plasma from 20 healthy subjects, n-alkane mix C9-C40 (GL sciences INC, Tokyo, Japan) containing decafluorotriphenylphosphine (DFTPP) (Sigma-Aldrich) were injected after every 5 study samples to monitor the stability of the analytical system. The QC samples were generated by mixing the same volume of plasma from 20 healthy subjects. It was confirmed that overall MS sensitivity was highly stable based on the relative intensity of each fragment of DFTPP. In addition, the RSD of intensity of ribitol (internal control) in all study samples and QC samples was 10.0%, indicating high intra and inter-day stability of the overall GC/MS measurement, including plasma extraction and derivatization steps.

The obtained GC/MS data were converted to Analysis Base File (ABF) format using ABF converter (https://www.reifycs.com/AbfConverter/index.html). Feature detection, spectra deconvolution, metabolite identification, and peak alignment were performed using MS-DIAL software ver. 2.72 (<http://prime.psc.riken.jp/Metabolomics_Software/MS-DIAL/index.html>.) [17]. Each metabolite was calibrated using LOWESS/Spline correction curve based on the QC values [18], after the substances for which residual standard deviation (RSD) of QC samples was above 40% were excluded from further analysis. As a result of these processes, the intra- and inter-day precision of the quantification of each metabolite used in this analysis was secured within a certain range, and in addition, the variability of quantification of each metabolite was corrected.

**Supplementary Figure 1**

Disposition of study subjects

**Supplementary Figure 2**

Association between the plasma levels of indoxyl sulfate, mannitol, mesoerythritol, and pyroglutamic acid, and baPWV in the first and the second datasets.


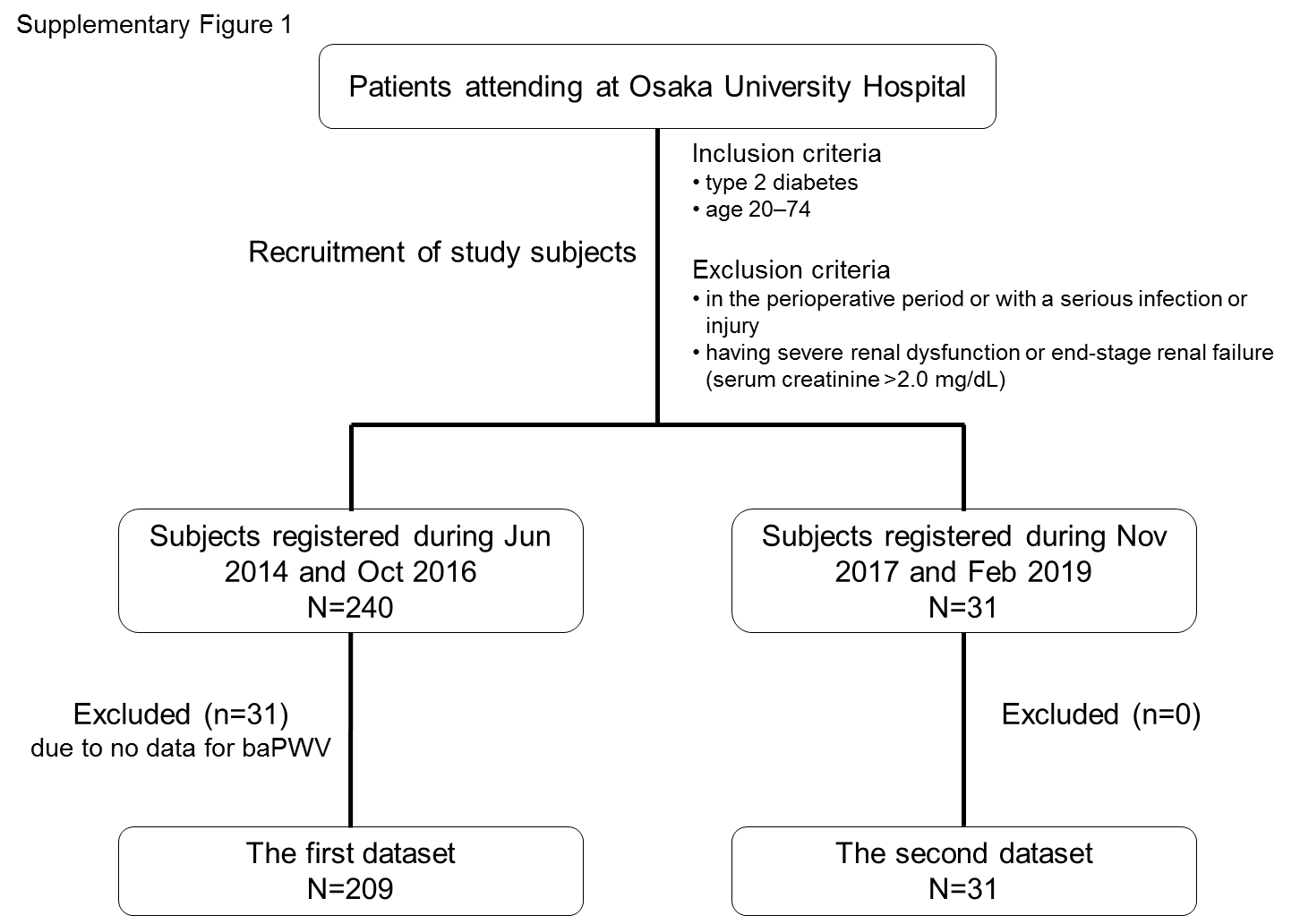


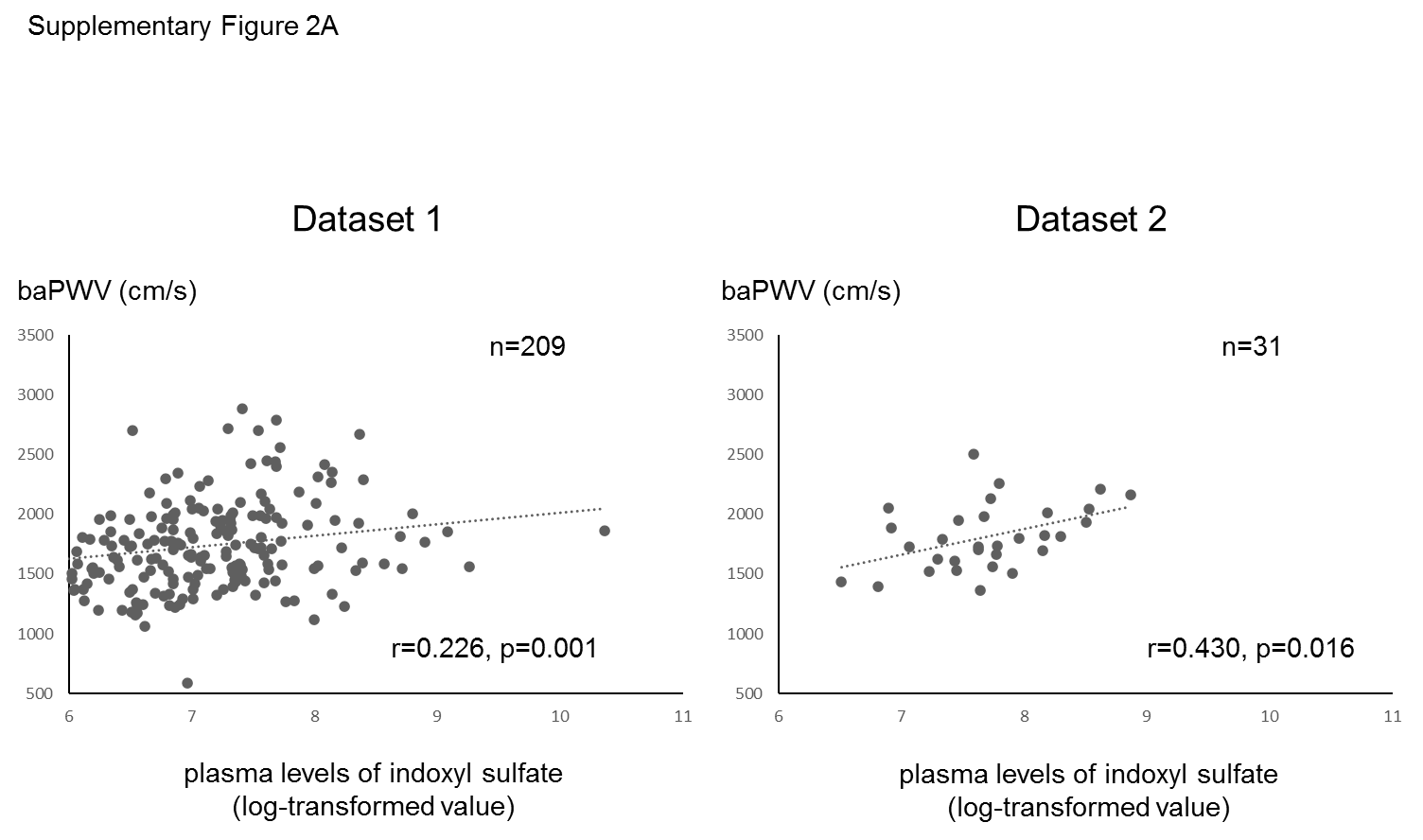


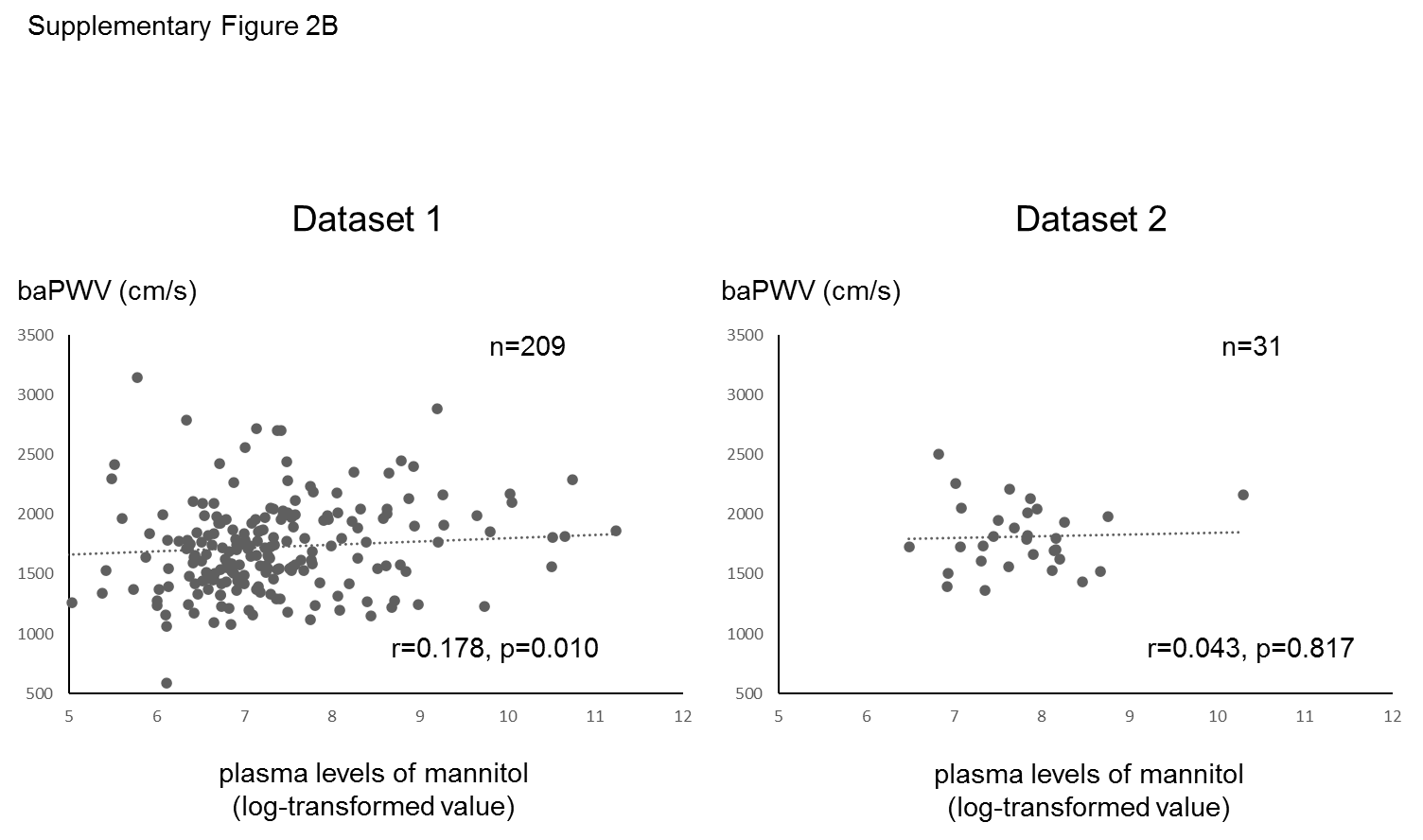


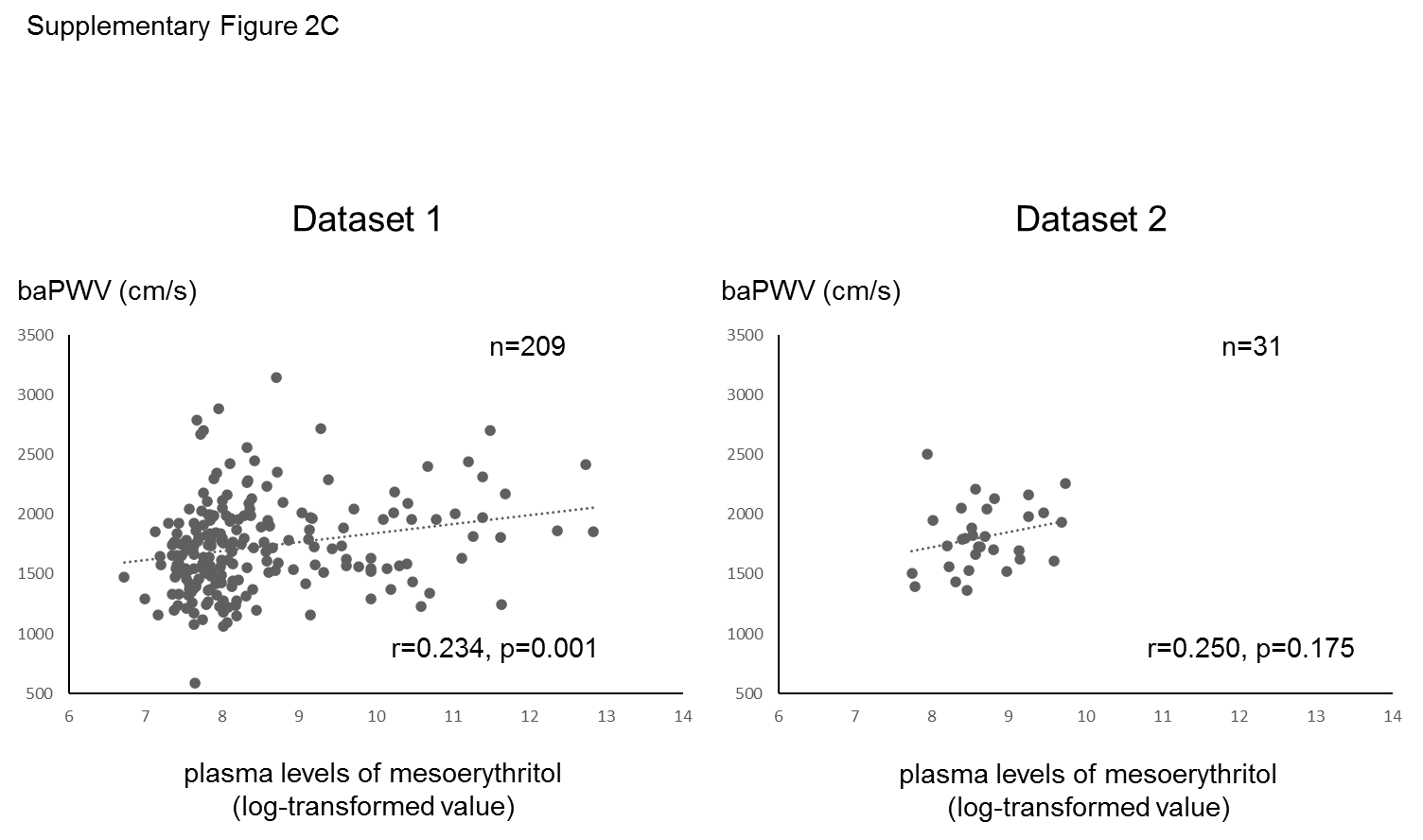


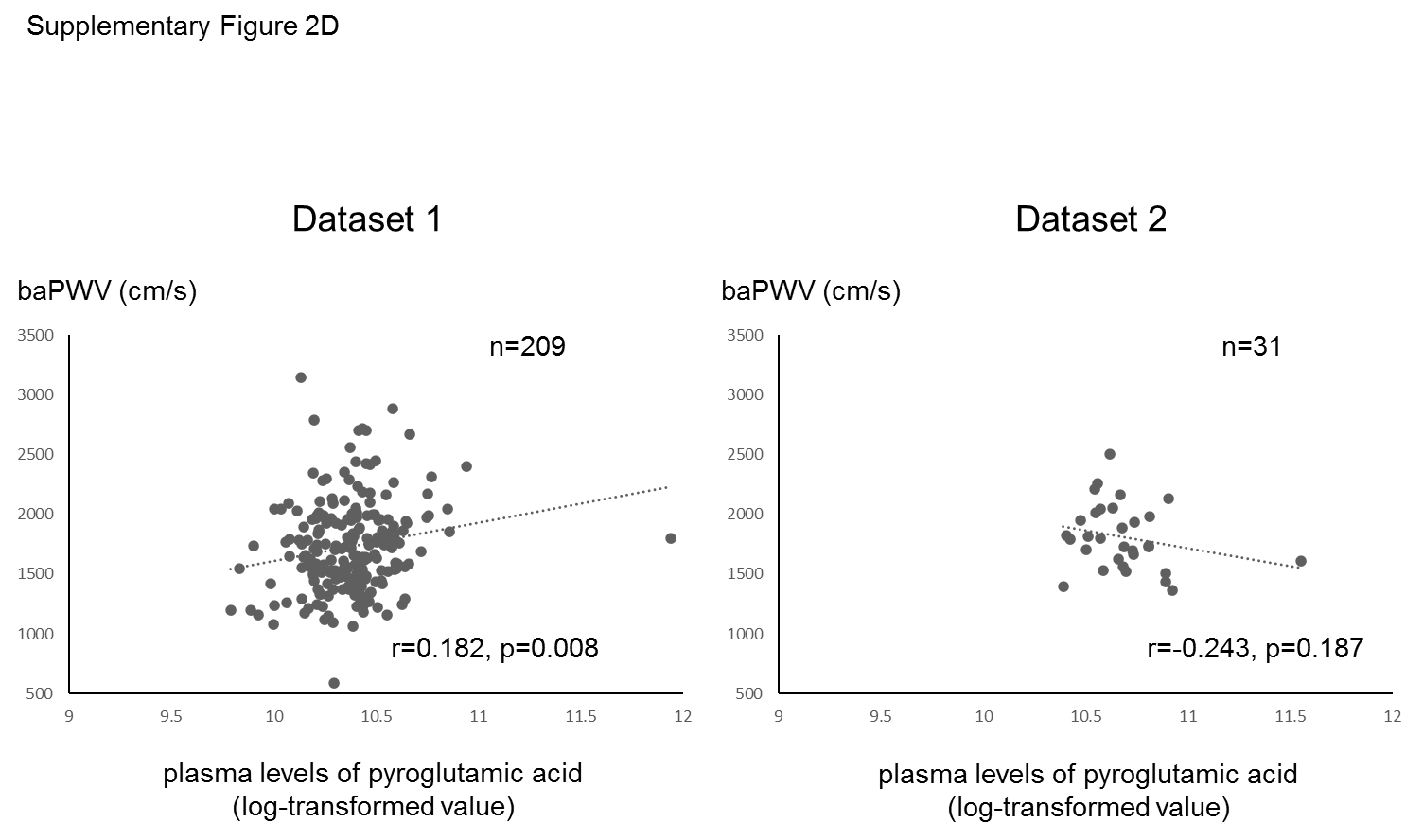

Supplement: Supplementary file 1 — Additional file 1. Figure S1. Disposition of study subjects. Figure S2. Association between the plasma levels of indoxyl sulfate, mannitol, mesoerythritol, and pyroglutamic acid, and baPWV in the first and the second datasets. [file 12933_2020_1057_MOESM1_ESM.docx]
